# Targeted release of budesonide modulates key gut microbial abnormalities in immunoglobulin A nephropathy

**DOI:** 10.3389/fimmu.2026.1856526

**Published:** 2026-08-07

**Authors:** Xiaoyi Mao, Da Shang, Sansi Chang, Haichun Yang, Lingyun Lai

**Affiliations:** 1 Division of Nephrology, Huashan Hospital, Fudan University, Shanghai, China; 2 Department of Pathology, Microbiology, and Immunology, Vanderbilt University Medical Center, Nashville, TN, United States

**Keywords:** budesonide, gut dysbiosis, gut microbiota, IgA nephropathy, targeted release

## Abstract

**Background:**

Gut microbiota dysbiosis has been implicated in the pathogenesis of immunoglobulin A nephropathy (IgAN), but whether different therapies are associated with distinct microbial profiles remains unclear. We aimed to investigate gut microbiota changes in IgAN patients receiving targeted-release budesonide (Nefecon), systemic glucocorticoids, or non-immunosuppressive therapy.

**Methods:**

In this comparative study, 24 biopsy-proven IgAN patients were divided into four groups: newly diagnosed untreated patients (n = 8), patients in remission after non-immunosuppressive therapy (n = 6), patients treated with Nefecon for at least 3 months (n = 5), and patients treated with systemic glucocorticoids for at least 3 months (n = 5). Nine healthy volunteers served as controls. Fecal microbiota was profiled by 16S rRNA gene sequencing, and community diversity, taxonomic composition, and associations with clinical/pathological parameters were analyzed.

**Results:**

Newly diagnosed IgAN patients exhibited reduced alpha diversity and a distinct beta diversity profile compared with healthy controls. The Nefecon group exhibited a microbial community structure more similar to that of controls than the newly diagnosed or remission groups. Several bacterial genera were significantly altered in newly diagnosed IgAN patients, including taxa enriched relative to both controls and the Nefecon group. Furthermore, microbiota composition correlated with renal pathological features, particularly segmental glomerulosclerosis and sclerosis ratio.

**Conclusion:**

Although limited by a small sample size, these findings suggest that targeted mucosal immunomodulation with Nefecon may partially restore gut microbial homeostasis in IgAN patients, providing new insight into the gut-kidney axis and the microbiota-related effects of therapy.

## 1 Introduction

Immunoglobulin A nephropathy (IgAN) remains the most prevalent primary glomerulonephritis globally, representing a leading cause of chronic kidney disease, particularly in Asian populations (1, 2). An 11-year analysis revealed that IgAN accounted for 44.40% of biopsy-proven primary glomerular nephropathy (PGN) cases, making it the most common primary glomerulonephritis (3). A hallmark of IgAN pathogenesis is the presence of galactose-deficient IgA1 (Gd-IgA1), an autoantigen generated by mucosal immune responses (4). These pathogenic Gd-IgA1 molecules form immune complexes that deposit in the glomerular mesangium, initiating complement activation and progressive renal injury (5).

Accumulating evidence implicates intestinal dysbiosis as an important environmental factor in IgAN pathogenesis (6). An altered gut microbiota is hypothesized to disrupt mucosal tolerance, promote the synthesis of aberrantly glycosylated IgA1, and perpetuate systemic autoimmunity (7). Crucially, fecal microbiota transplantation (FMT) from IgAN patients into susceptible mice recapitulates key disease features, including elevated Gd-IgA1 levels and renal IgA deposition (8). This evidence supports a causal role for gut microbiota in IgAN, moving beyond mere association.

While the role of the microbiome in disease initiation is increasingly recognized, the impact of therapeutic interventions on this pathogenic microbial community has not been fully elucidated. Existing studies have primarily consisted of cross-sectional studies comparing the microbiota profiles between IgAN patients and healthy controls (9, 10). A limited number of investigations have examined changes following broad-spectrum immunosuppression, but the systemic effects of agents like glucocorticoids complicate the interpretation of their targeted interactions with the gut microbiota (1). Furthermore, it remains unknown whether clinical remission achieved with the non-immunosuppressive therapy of renin-angiotensin system (RAS) blockade correlates with a resolution of the underlying gut dysbiosis linked to IgAN pathogenesis.

Nefecon, a targeted-release formulation of budesonide designed for delivery to the ileocecal region, acts as a mucosa-targeted immunomodulator on this primary site of gut-associated lymphoid tissue (11). This targeted action enables the investigation of whether specific modulation of the mucosal immune environment can reverse IgAN-associated dysbiosis. However, the specific effects of Nefecon on the gut microbiota in IgAN patients are unknown. It is also unclear whether any induced microbial changes are synchronous with clinical improvement or represent a distinct biological effect of therapy.

To address these questions, we compared the gut microbiota of healthy volunteers and IgAN patients across distinct clinical states: newly diagnosed, in remission after non-immunosuppressive therapy, actively treated with systemic glucocorticoids without complete remission, and following a 3-month course of Nefecon also without complete remission. This study aimed to elucidate the unique microbial shifts induced by Nefecon, providing novel insights into the relationship between targeted mucosal therapy and the gut microbiome in IgAN.

## 2 Methods

### 2.1 Study design

This study enrolled adult patients with biopsy-proven IgAN who were treated at the Division of Nephrology, Huashan Hospital between November 2023 and November 2024, along with healthy volunteers. All participants provided fecal samples. Patients with IgAN were divided into four groups: (1) newly diagnosed IgAN patients at the time of renal biopsy with concurrent fecal sample collection(new patients group); (2) the non-immunosuppressive therapy-induced remission group (remission group), defined as a 24-hour urine protein level < 0.4 g with stable renal function; (3) the Nefecon-treated group (Nefecon group), consisting of patients who had received Nefecon (16 mg once daily) for at least three months at the time of fecal sample collection without attaining complete remission; and (4) the glucocorticoids-treated group (steroid group), comprising patients receiving oral prednisone or methylprednisolone for at least three months at fecal sample collection, also without complete remission. Healthy volunteers served as the healthy control group. The exclusion criteria for all participants were as follows: (1) secondary IgAN; (2) end-stage renal disease (ESRD); (3) antibiotic use within one month prior to sample collection; (4) a history of gastrointestinal diseases; and (5) active infection.

### 2.2 Clinical and biochemical assessments

24-hour urine protein was measured by turbidimetry using a Roche kit and analyzer. Serum creatinine was measured using an enzymatic method on a Roche platform. The estimated glomerular filtration rate (eGFR) was calculated using the Chronic Kidney Disease Epidemiology Collaboration (CKD-EPI) equation. Renal pathological lesions were evaluated according to the Oxford classification (MEST-C score), and the percentage of global glomerulosclerosis was calculated based on light microscopy examination of renal biopsy specimens.

### 2.3 Sample collection and DNA extraction

Fecal samples were collected using sterile containers and immediately stored at -80°C in DNA preservation solution. Genomic DNA was extracted using the TGuide S96 Magnetic Soil/Stool DNA Kit (Tiangen Biotechnology, Beijing, China) according to the manufacturer’s instructions.

### 2.4 Gene sequencing

The full-length 16S rRNA gene was amplified from the extracted DNA using the forward primer 27F (5’-AGRGTTTGATYNTGGCTCAG-3’) and reverse primer 1492R (5’-TASGGHTACCTTGTTASGACTT-3’). Polymerase chain reaction (PCR) was performed in a 10 μL reaction volume. The amplicons were quantified, pooled at equimolar concentrations, and sequenced on the PacBio Sequel II platform (Beijing Biomarker Technologies Co., Ltd., Beijing, China). Bioinformatic processing included demultiplexing based on barcodes and quality control of the generated circular consensus sequencing (CCS) reads.

### 2.5 Statistical analysis

Operational taxonomic units (OTUs) were clustered from the qualified sequences at a 97% similarity threshold using USEARCH (version 10.0). Taxonomy was assigned to OTUs using the Naive Bayes classifier in QIIME2 against the SILVA database (release 138.1) with a 70% confidence threshold. Alpha (α)-diversity was calculated using the Shannon and Simpson indices in QIIME2 to evaluate the species diversity within each sample. Beta (β)-diversity was analyzed via principal coordinate analysis (PCoA) and non-metric multidimensional scaling (NMDS) to assess the compositional differences between samples. The Kruskal-Wallis test was employed to compare bacterial abundance and diversity among groups. Pairwise comparisons were performed using the Nemenyi post-hoc test to control for family-wise error rate when significant differences were detected. Differentially abundant taxa were identified using linear discriminant analysis effect size (LEfSe). Furthermore, to compare our results with genera previously reported in IgAN studies (7, 10, 12–21), supplementary pairwise analyses using the Mann-Whitney U test were conducted. Pearson correlation analysis was performed to assess the relationships between clinical/pathological characteristics and the gut microbiota. Associations in species abundance across samples were assessed using Spearman rank correlation. The relationship between the microbiota and the clinical/pathological data was further visualized using distance-based redundancy analysis (dbRDA).

## 3 Results

### 3.1 Participant characteristics

**Figure 1:**
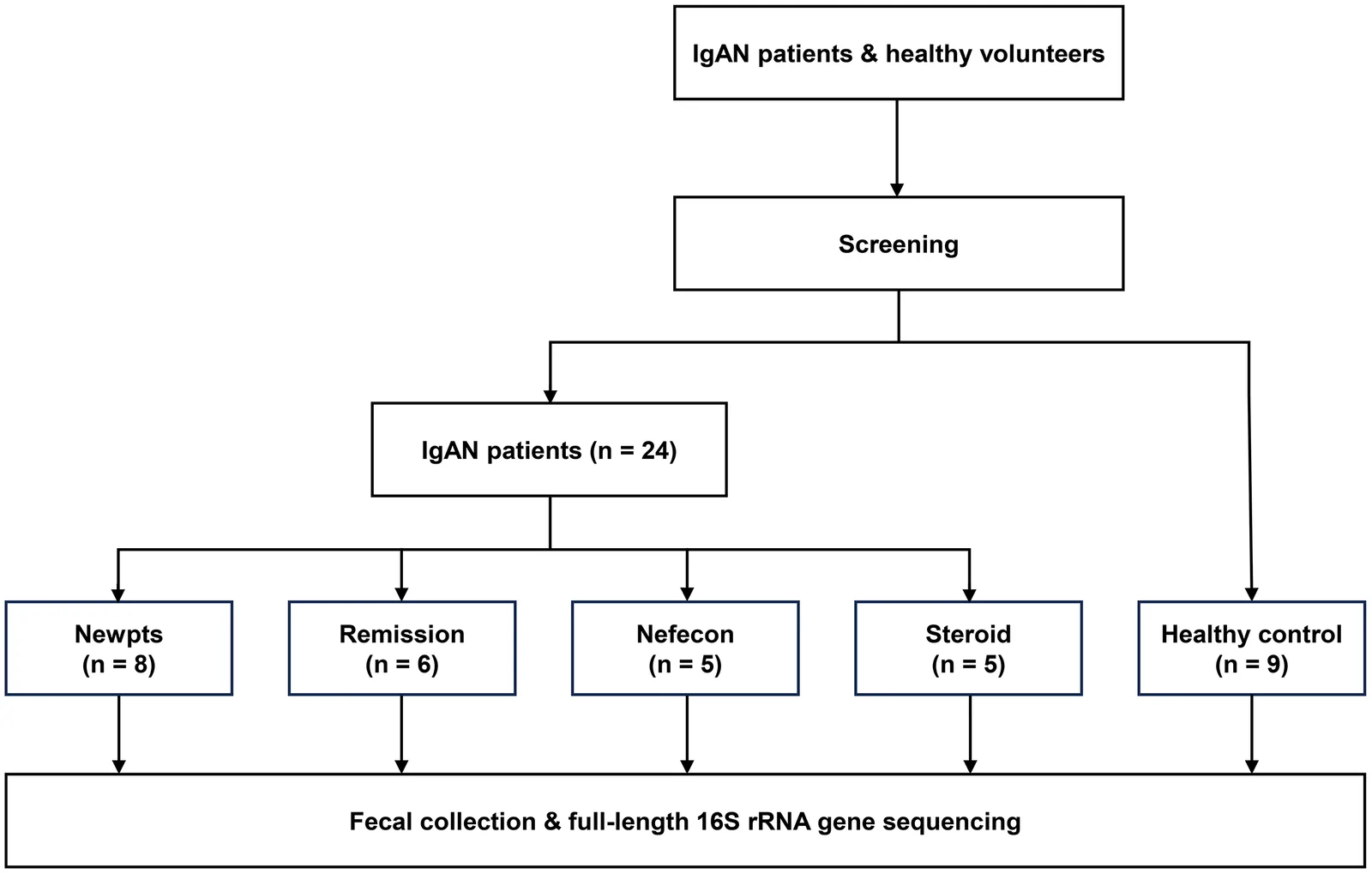
Study design. Newpts, new patients.

**Figure 2:**
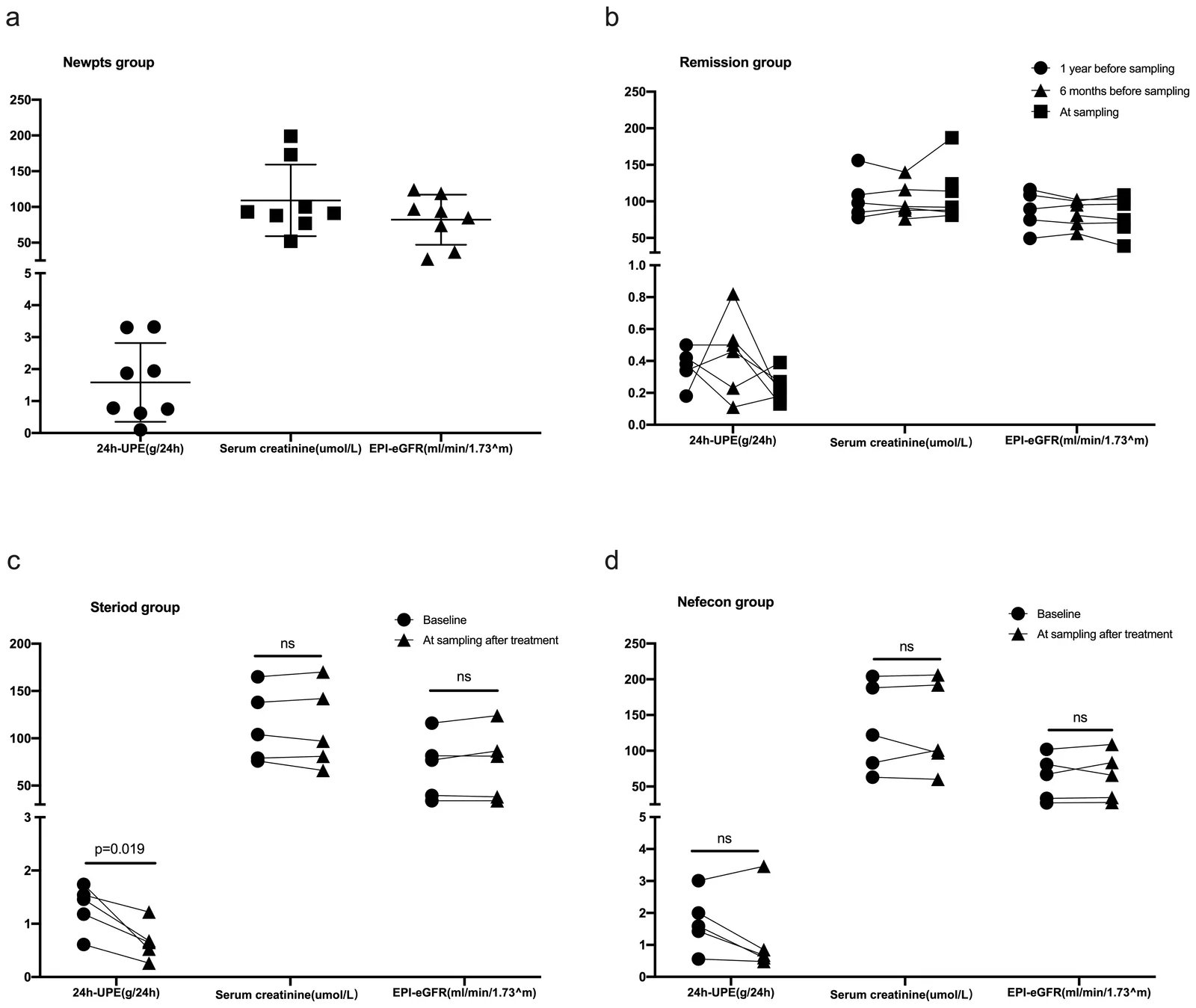
Clinical characteristics of participants in different groups. **(a)** 24-hour urine protein, serum creatinine and eGFR of the new patients group. Data are presented as mean ± SD (standard deviation). **(b)** 24-hour urine protein, serum creatinine and eGFR of the remission group 1 year and 6 months prior to, and at, sampling. **(c)** 24-hour urine protein, serum creatinine and eGFR of the steroid group at baseline and after treatment. **(d)** 24-hour urine protein, serum creatinine and eGFR of the Nefecon group at baseline and after treatment. Newpts, new patients. 24h-UPE, 24 hour-urine protein excretion.

A total of 33 participants were enrolled in this study, comprising 8 in the new patients group, 6 in the remission group, 5 in the Nefecon group, 5 in the steroid group, and 9 in the healthy control group (**Figure 1**). Detailed clinical information for every IgAN patient is provided in **Supplementary Table 1**. In the new patients group, the 24-hour urine protein was 1.59 ± 1.23 g, with a serum creatinine level of 109.1 ± 50.1 μmol/L and an eGFR of 82.1 ± 35.1 mL/min/1.73 m2 (**Figure 2a**, data are expressed as mean ± SD). Patients in the remission group, at the time of sample collection, exhibited 24-hour urine protein levels below 0.4 g, with relatively stable serum creatinine and eGFR values (**Figure 2b**). Regarding therapeutic outcomes, the steroid group demonstrated a significant reduction in 24-hour urine protein from 1.31 ± 0.44 g at baseline to 0.67 ± 0.35 g after treatment (p=0.019, paired t-test, data are expressed as mean ± SD), although no significant changes were observed in serum creatinine or eGFR (**Figure 2c**). In the Nefecon group, 24-hour urine protein decreased from 1.72 ± 0.89 g to 1.21 ± 1.27 g after three months of treatment, though this change was not statistically significant (p=0.169, paired t-test, data are expressed as mean ± SD). Serum creatinine and eGFR levels also remained stable throughout the treatment period in this group (**Figure 2d**).

### 3.2 Characteristics of gut microbiota across groups

**Figure 3:**
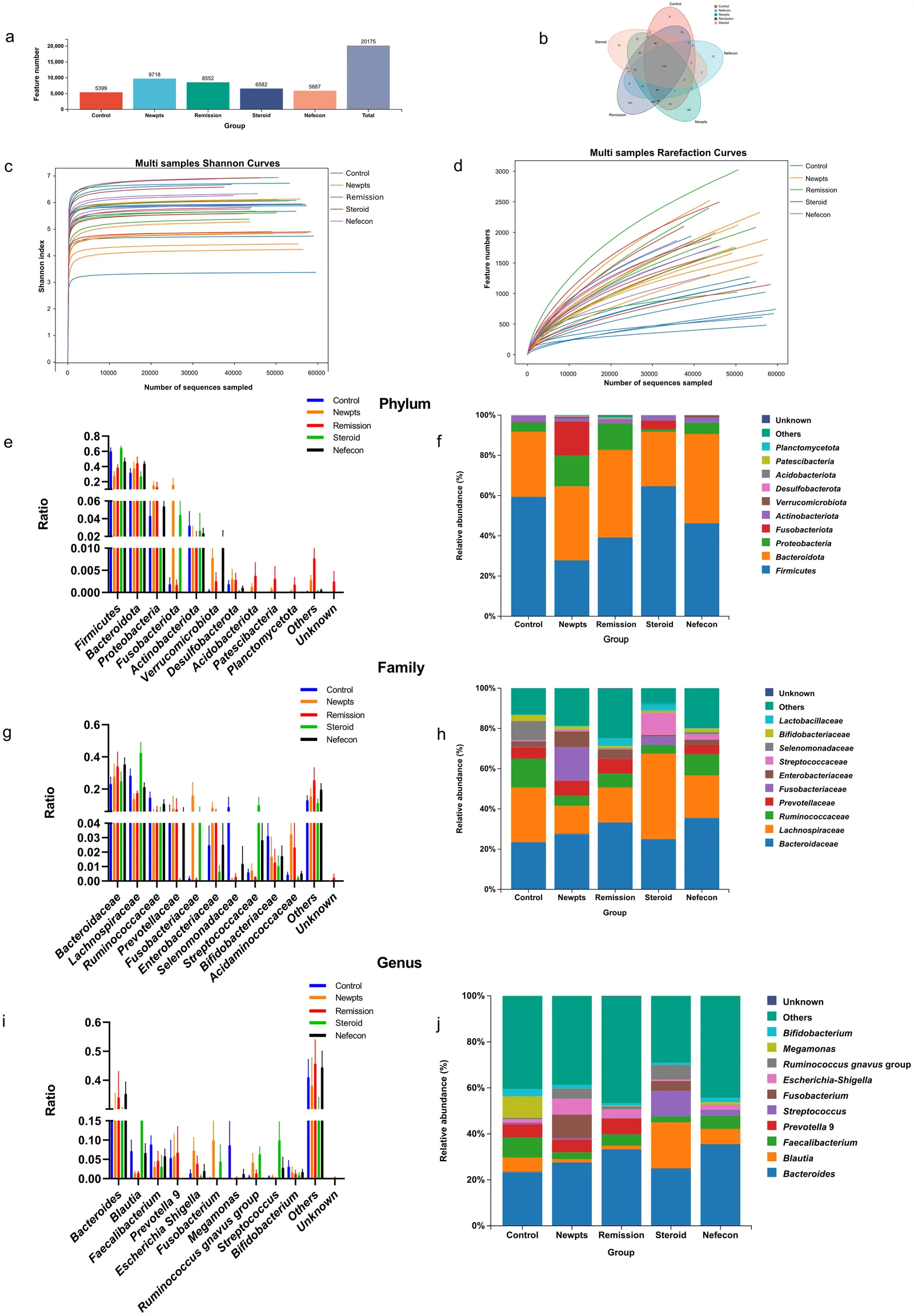
Characteristics of gut microbiota across groups. **(a)** Feature number of each group. **(b)** Venn diagram of features. **(c)** Shannon index curve. **(d)** Rarefaction curve. **(e)** Relative abundance of the top ten phyla. **(f)** Stacked bar plots of the top ten phyla. **(g)** Relative abundance of the top ten families. **(h)** Stacked bar plots of the top ten families. **(i)** Relative abundance of the top ten genera. **(j)** Stacked bar plots of the top ten genera. Newpts, new patients.

Analysis of 16S rRNA gene sequencing data yielded 20,175 effective OTUs across all participants. The distribution of OTUs was as follows: 5,399 in the healthy control group; 9,718 in the new patients group; 8,522 in the remission group; 6,582 in the steroid group, and 5,887 in the Nefecon group (**Figure 3a**). A Venn diagram illustrated 151 genera common to all five groups (**Figure 3b**). Both Shannon and Rarefaction curves revealed that the sequencing depth was sufficient to capture the majority of microbial diversity, as curves approached saturation in all samples (**Figures 3c, d**).

A total of 1171 genera across 39 phyla were identified in the fecal samples (**Supplementary Table 2**). Distinct microbial compositional patterns were observed at different taxonomic levels among the groups. At the phylum level, *Firmicutes* predominated in the healthy control, steroid, and Nefecon groups. In contrast, the new patients and remission groups showed increased relative abundances of *Proteobacteria* and *Desulfobacterota*. *Fusobacteriota* was notably abundant in the new patients and steroid groups, whereas *Bacteroidota* and *Actinobacteriota* remained relatively consistent across all five groups. The remission group also displayed a higher abundance of *Acidobacteriota*, *Patescibacteria*, and *Planctomycetota* (**Figures 3e, f**). Family-level analysis revealed high abundances of *Ruminococcaceae*, *Selenomonadaceae*, and *Bifidobacteriaceae* in the healthy control and Nefecon groups, whereas *Fusobacteriaceae*, *Enterobacteriaceae*, and *Acidaminococcaceae* were less prevalent. An increased relative abundance of *Streptococcaceae* was observed in the steroid and Nefecon groups (**Figures 3g, h**). At the genus level, new patients and remission groups exhibited lower abundances of *Blautia*, *Faecalibacterium*, and *Bifidobacterium* compared to other groups, alongside a rising trend in *Escherichia-Shigella*. Notably, *Megamonas* was identified as a predominant genus in IgAN patients across all disease groups, regardless of treatment status (**Figures 3i, j**).

### 3.3 Similar microbial diversity in Nefecon and healthy control groups

**Figure 4:**
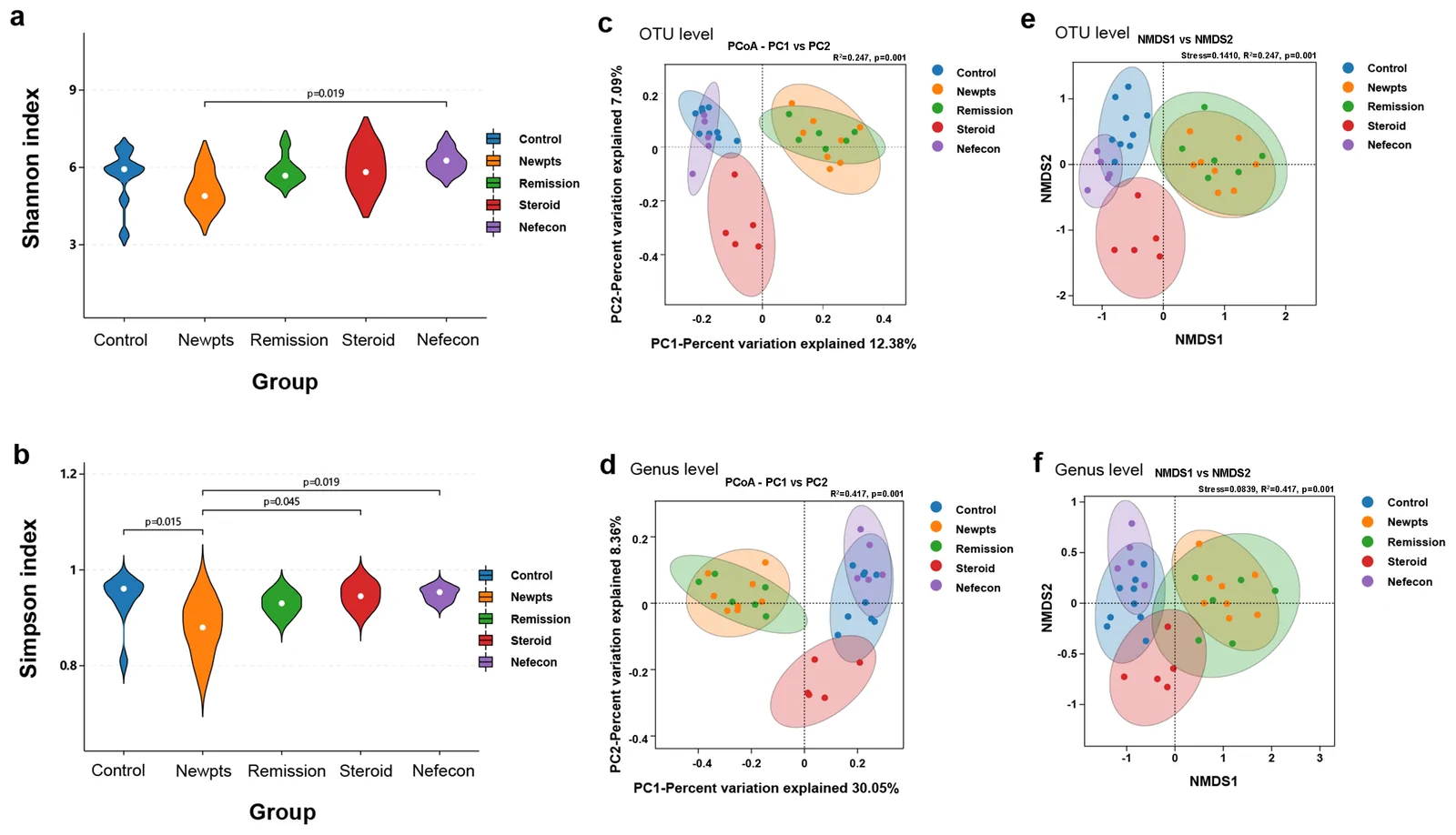
Comparison of alpha (α) and beta (β) diversity between groups. **(a)** α-diversity measured by the Shannon index. **(b)** α-diversity measured by the Simpson index. **(c)** β-diversity assessed by principal coordinate analysis (PCoA) based on unweighted UniFrac distance at the OTU level. **(d)** β-diversity assessed by PCoA at the genus level. **(e)** β-diversity assessed by non-metric multidimensional scaling (NMDS) at the OTU level. **(f)** β-diversity assessed by NMDS at the genus level. Newpts, new patients.

Analysis of α-diversity demonstrated that in the Shannon index (**Figure 4a**), the Nefecon group exhibited higher α-diversity than the new patients group (p=0.019), and a similar trend toward higher diversity was observed in the healthy control group. Similarly, the Simpson index (**Figure 4b**) showed significantly higher α-diversity in the healthy control and Nefecon groups compared to the new patients group (p=0.015 and p=0.019, respectively). A trend toward lower diversity was observed in the new patients and remission groups in the ACE and Chao1 indices. No significant difference in α-diversity was detected between the Nefecon and steroid groups across all indices.

Analysis of β-diversity revealed that the microbial community structures of the healthy control and Nefecon groups were similar in both PCoA (**Figures 4c, d**) and NMDS (**Figures 4e, f**) analyses at the OTU (Stress=0.1410, R2 = 0.247, p=0.001) and genus levels (Stress=0.0839, R2 = 0.417, p=0.001). The steroid group also showed a structural trend closer to the healthy control and Nefecon groups, while the remission group clustered nearer to the new patients group.

### 3.4 Differences in microbial communities among groups

**Figure 5:**
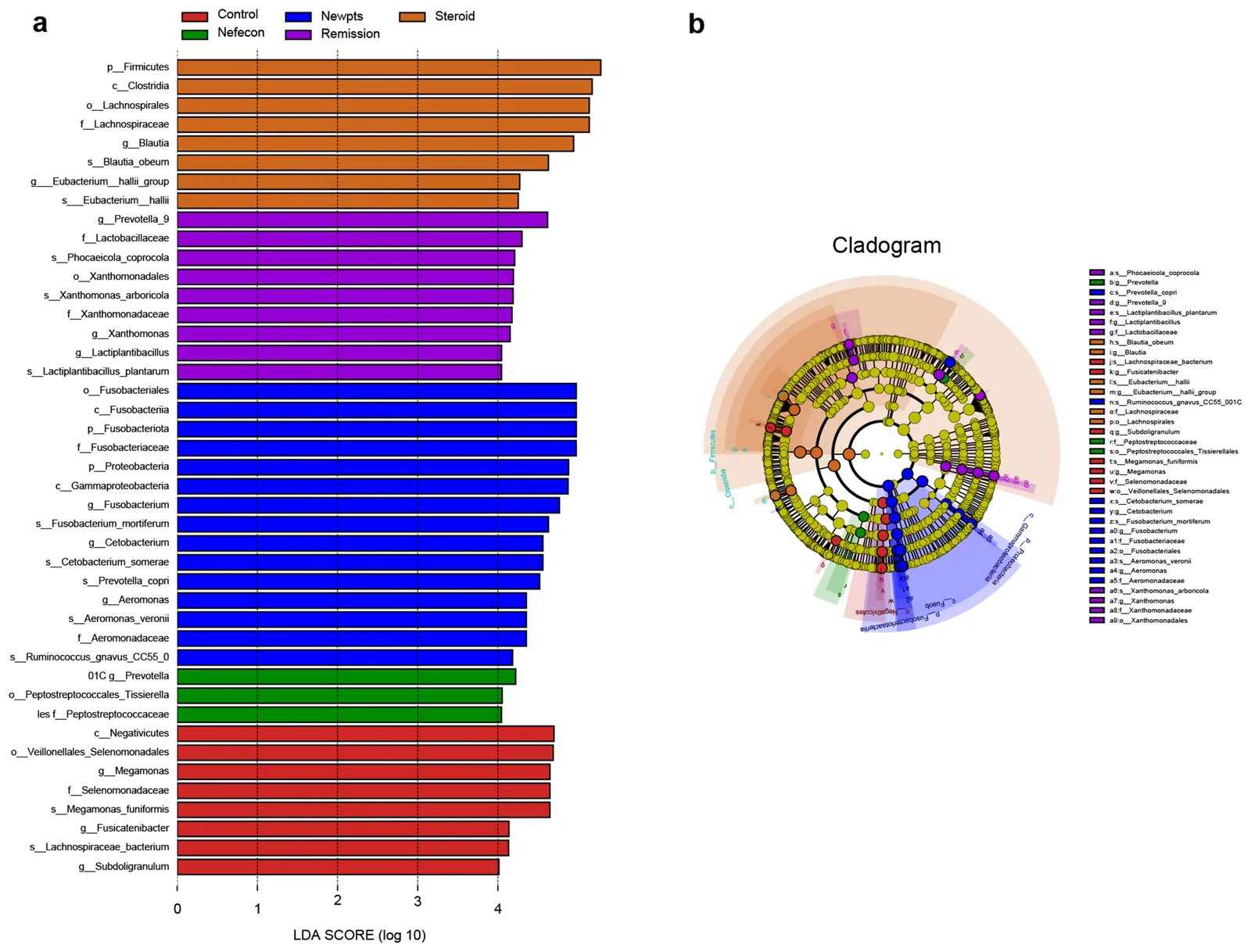
Linear discriminant analysis effect size (LEfSe) between groups. **(a)** Linear discriminant analysis (LDA) score distribution histogram. Y-axis: differentially abundant taxa; X-axis: log10 LDA score. A longer bar indicates a more significant difference. **(b)** Cladogram from LEfSe analysis. Circles from center to outward represent taxonomic levels from phylum to species. Node size indicates relative abundance. Nodes are colored according to the group with the highest abundance. Newpts, new patients.

**Figure 6:**
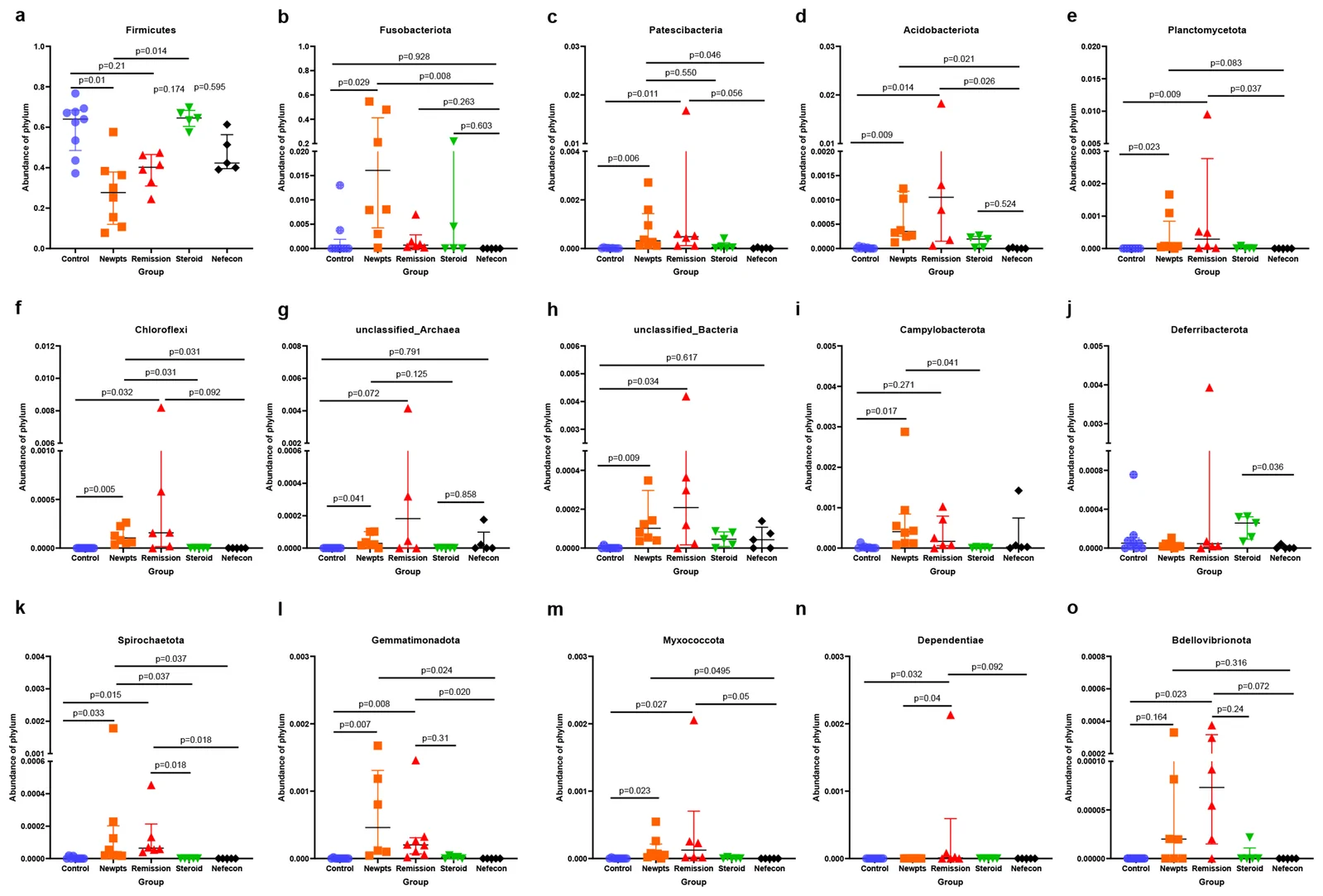
Differential phyla among all groups. Relative abundance of **(a)** *Firmicutes*, **(b)** *Fusobacteriota*, **(c)** *Patescibacteria*, **(d)** *Acidobacteriota*, **(e)** *Planctomycetota*, **(f)** *Chloroflexi*, **(g)** unclassified Archaea, **(h)** unclassified Bacteria, **(i)** *Campylobacterota*, **(j)** *Deferribacterota*, **(k)** *Spirochaetota*, **(l)** *Gemmatimonadota*, **(m)** *Myxococcota*, **(n)** *Dependentiae*, **(o)** *Bdellovibrionota*. The horizontal lines represent the medians, and the error bars represent the interquartile ranges (IQRs). Kruskal-Wallis test was used to compare bacterial abundance and diversity among groups, followed by Nemenyi *post-hoc* test for pairwise comparisons. Newpts, new patients.

Given the significant β-diversity differences among the groups, LEfSe linear discriminant analysis was performed, revealing a unique microbial signature for each group. The genus *Prevotella*, the orders *Peptostreptococcales Tissierellales*, and the family *Peptostreptococcaceae* were the most distinct microbial taxa identified in Nefecon group (**Figure 5**). Among the 39 phyla identified, 15 showed statistically significant differences among groups (**Figure 6**). *Firmicutes* was the most abundant phylum overall, with its relative abundance significantly elevated in the steroid and the healthy control groups, and showing an increasing trend in the Nefecon group. Compared to the new patients group, the Nefecon and healthy control groups had significantly lower abundances of *Fusobacteriota*, *Patescibacteria*, *Acidobacteriota*, *Chloroflexi*, *Spirochaetota*, *Gemmatimonadota* and *Myxococcota*. A similar reduction in *Acidobacteriota*, *Spirochaetota*, *Gemmatimonadota* and *Myxococcota* was observed in the Nefecon and healthy control groups relative to the remission group. The steroid group exhibited lower levels of *Chloroflexi*, *Campylobacterota*, and *Spirochaetota* than the new patients group, partially aligning with the profiles of the healthy control and Nefecon groups.

**Figure 7:**
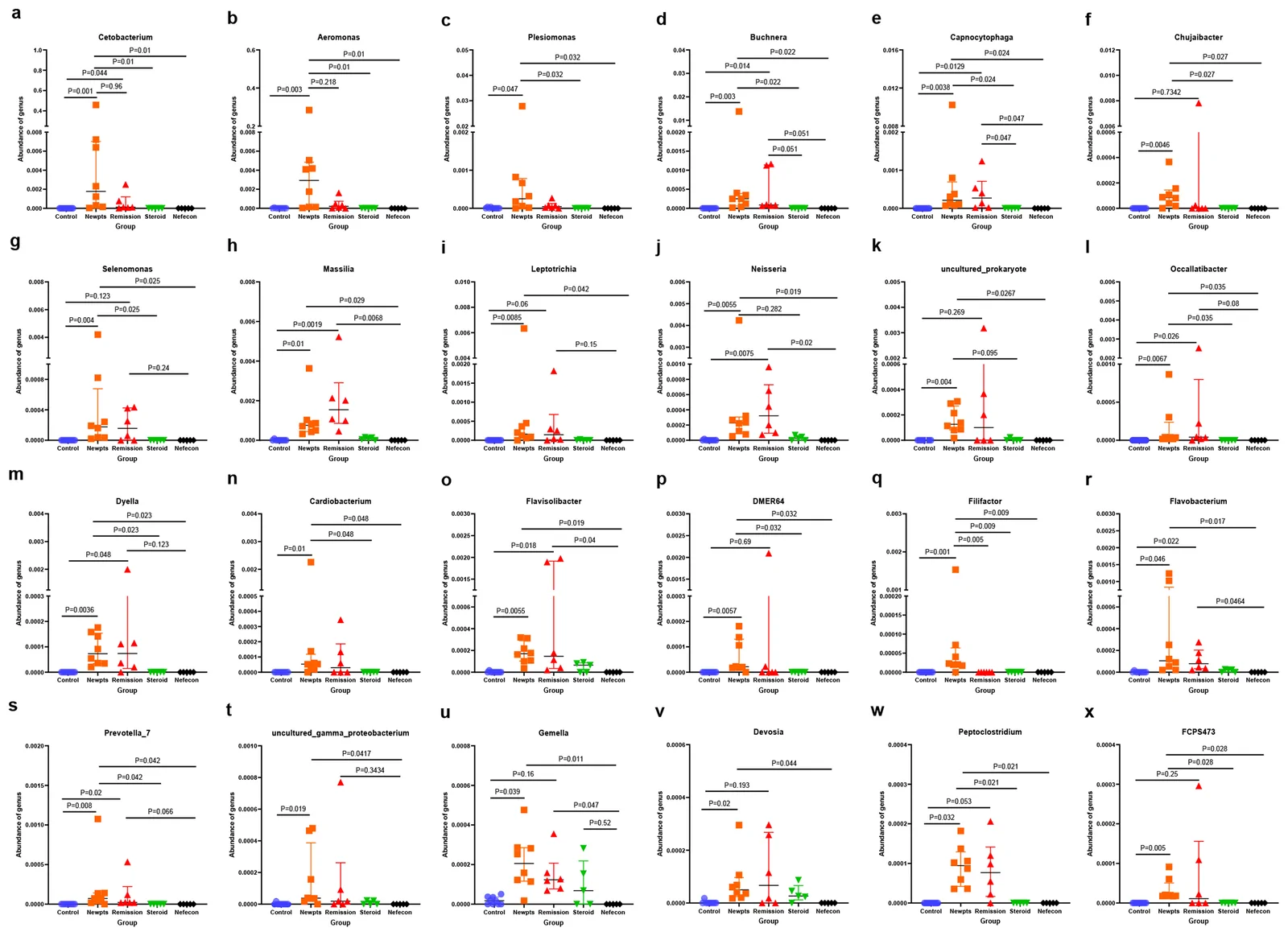
Genera exhibiting differential abundance both between new patients and healthy control groups and between new patients and Nefecon groups. Relative abundance of **(a)** *Cedecea*, **(b)** *Aeromonas*, **(c)** *Plesiomonas*, **(d)** *Buchnera*, **(e)** *Capnocytophaga*, **(f)** *Chujibacter*, **(g)** *Solenomonas*, **(h)** *Massilia*, **(i)** *Leptotrichia*, **(j)** *Neisseria*, **(k)** uncultured prokaryote, **(l)** *Occallatibacter*, **(m)** *Dyella*, **(n)** *Cardiobacterium*, **(o)** *Flavisolibacter*, **(p)** OMER64, **(q)** *Filifactor*, **(r)** *Flavobacterium*, **(s)** *Prevotella* 7, **(t)** uncultured gamma proteobacterium, **(u)** *Gemella*, **(v)** *Devosia*, **(w)** *Peptoclostridium*, **(x)** FCP5473. The horizontal lines represent the medians, and the error bars represent the interquartile ranges (IQRs). Kruskal-Wallis test was used to compare bacterial abundance and diversity among groups, followed by Nemenyi post-hoc test for pairwise comparisons. Newpts, new patients.

A total of 132 differentially abundant genera were identified across the five groups (**Supplementary Table 3**). Among these, 24 genera were consistently more abundant in the new patients group than in both the healthy control and Nefecon groups (**Figure 7**). *Cetobacterium* and *Aeromonas* were the most dominant genera in the new patients group, with abundances significantly higher than those in the healthy control, steroid, and Nefecon groups, but were not significantly different from those in the remission group. Notably, only *Filifactor* exhibited a decrease in the remission group compared to the new patients group (**Figure 7q**). Beyond these 24 genera, an additional 9 genera exhibited significant differences between the new patients and Nefecon groups (**Supplementary Figure 1**).

Genera previously implicated in IgAN were next assessed. Among the 47 literature-reported genera detected in our study (**Supplementary Figures 2**, **3**), *Fusicatenibacter*, *Romboutsia*, *Dorea*, *Anaerostipes*, *Faecalibacterium*, *Coprococcus* and *Lachnospiraceae* UCG 004 were significantly decreased in new patients group compared to healthy control group, while *Sphingomonas*, *Prevotella* and *Ralstonia* were increased. Notably, for most of these genera, no significant differences were observed between the healthy control and Nefecon groups, except for *Ralstonia*, which was elevated following Nefecon treatment.

### 3.5 Association between clinical/pathological characteristics and the gut microbiota

**Figure 8:**
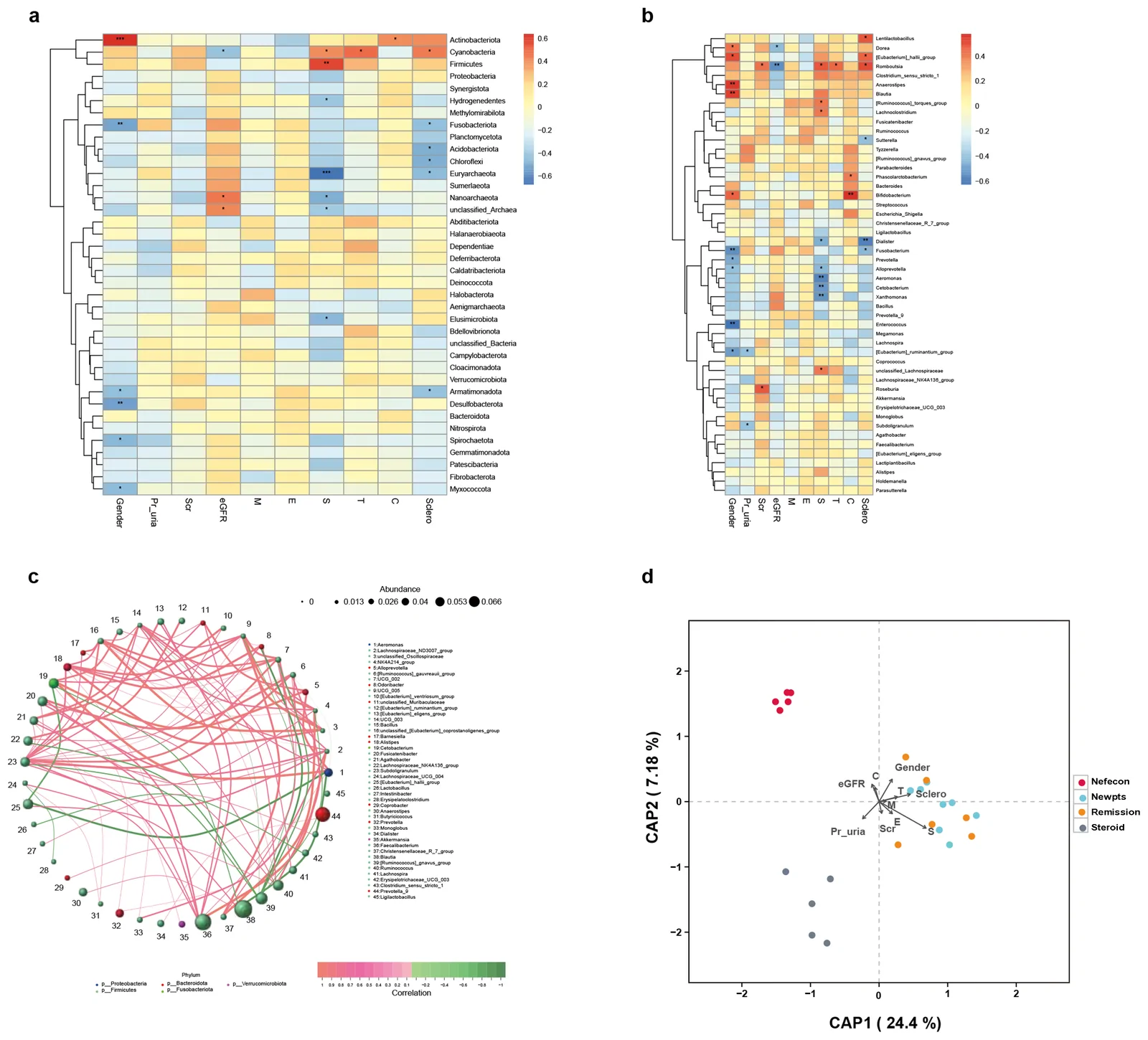
Correlation analysis between gut microbiota and clinical/pathological characteristics. **(a)** Heatmap of Pearson’s correlation coefficients between the top 50 abundant phyla and clinical/pathological indices. **(b)** Heatmap of Pearson’s correlation coefficients between the top 50 abundant genera and clinical/pathological indices. Colors represent Pearson correlation coefficients, with red indicating negative correlation, white indicating no correlation, and blue indicating positive correlation. Significant correlations are marked with asterisks (*p < 0.05, **p < 0.01, ***p < 0.001). **(c)** Correlation network of microbiota at the genus level based on Spearman’s correlation analysis. Circle size represents abundance. Edge thickness represents correlation strength, with red lines indicating positive correlations and green lines indicating negative correlations. **(d)** Distance-based redundancy analysis (dbRDA) illustrating the relationship between microbiota composition and clinical/pathological data. Newpts, new patients; Sclero, percentage of global glomerulosclerosis; C, crescent; T, interstitial fibrosis/tubular atrophy; S, segmental sclerosis; E, endocapillary cellularity; M, mesangial hypercellularity; eGFR, estimated glomerular filtration rate; Scr, serum creatinine; Pr_uria, 24-hour urine protein.

To explore the relationship between gut microbiota and disease manifestations in IgAN, Pearson correlation analysis between the top 50 abundant phyla and genera and key clinical and pathological indices, including demographic data, renal pathology, 24-hour urine protein, and serum creatinine, was performed (**Figures 8a, b**). At both the phylum and genus levels, the gut microbiota showed limited correlation with clinical measures such as 24-hour urine protein and serum creatinine. In contrast, marked associations were observed with renal pathological lesions, particularly segmental glomerulosclerosis and the glomerulosclerosis ratio. Several microbial taxa were significantly correlated with these pathological indices, showing either positive or negative relationships (**Figures 8a, b**). Additionally, *Aeromonas*, *Cetobacterium*, *Fusicatenibacter*, *Faecalibacterium*, *Blautia*, *Ruminococcus gnavus* group, and *Prevotella* 9 were among the most abundant microbial taxa detected across participants. The connecting lines between taxa indicate the strength of their negative or positive correlations (**Figure 8c**).

dbRDA revealed that the first two constrained axes accounted for a substantial portion of the variance in microbiota composition explained by clinical/pathologic variables (CAP1 = 24.4%, CAP2 = 7.18%, cumulative ~31.6%, **Figure 8d**). CAP1 clearly separated the samples into two main clusters: samples from Nefecon and steroid groups were predominantly distributed on the left side of the ordination, while those from the new patients and remission groups clustered on the right. This indicates that CAP1 primarily reflects differences in gut microbial composition associated with treatment or disease status, specifically separating the Nefecon/steroid groups from the new patients/remission groups. CAP2 provided further separation along the vertical axis, distinguishing Nefecon samples (upper-left) from steroid samples (lower-left). Overall, Nefecon samples formed a relatively compact cluster in the upper-left region, suggesting within-group similarity. Steroid samples clustered in the lower-left, and new patients and remission samples showed overlap on the right side of the plot, indicating greater similarity between these two groups along the constrained axes.

## 4 Discussion

IgA nephropathy is increasingly recognized as an autoimmune disorder, and emerging evidence suggests that intestinal dysbiosis may contribute to the production of pathogenic Gd-IgA1, although the exact mechanisms remain under investigation (4, 5, 7). In this study, we investigated the gut microbiota in IgAN patients under different clinical conditions. Our primary finding is that Nefecon, a targeted-release formulation of budesonide, appeared to induce shifts in the gut microbiota, making it more closely resemble that of healthy controls. Interestingly, this effect was not observed in patients who achieved clinical remission with RAS blockade alone, suggesting a potential disconnection between gut microbial ecology and clinical proteinuria in the short term. Consistent with this observation, a recent study suggested that clinical parameters alone may not fully capture treatment responses in IgAN patients, whereas gut microbial features showed promising predictive potential (22).

Greater similarity in the overall structure of the gut microbiota (β-diversity) was observed between the Nefecon group and the healthy control group. This observation may be attributed to the localized drug delivery mechanism of Nefecon, which targets the ileocecal region, a key site of gut-associated lymphoid tissue (23). By suppressing local mucosal immune responses in a targeted manner, Nefecon may reduce the production of Gd-IgA1 and alleviate intestinal inflammation (24), thereby potentially creating a more favorable environment for the restoration of a balanced gut microbiota. In contrast, glucocorticoids, while effective in reducing proteinuria, exert broader immunosuppressive effects that may not specifically rectify the mucosal immune dysregulation in the gut, resulting in a less complete normalization of the microbial community. This is consistent with the findings of a randomized controlled trial in which systemic immunosuppression with glucocorticoids failed to improve long-term renal outcomes in IgAN patients despite reducing proteinuria (25). Furthermore, patients in the remission group, despite having well-controlled proteinuria with RAS blockers, maintained a gut microbiota profile that closely resembled that of newly diagnosed, untreated patients. This indicates that the gut dysbiosis associated with IgAN may persist even after clinical remission is achieved. This finding also implies that the beneficial effect of Nefecon on the gut microbiota could be independent of its impact on proteinuria in the short term, potentially contributing to its long-term renal protective effects as reported in clinical trials (26). This interpretation is further supported by the phase 3 NefIgArd trial, which demonstrated that a 9-month course of Nefecon significantly attenuated eGFR decline over 2 years and delayed the time to confirmed 30% reduction in eGFR or kidney failure (11).

Our results at the genus level offered more detailed support for the observed microbial shifts. 24 bacterial genera were significantly more abundant in the new patients group compared to both the healthy control and Nefecon groups. This enrichment of specific taxa in untreated patients aligns with previous studies documenting a dysbiotic gut microbiome in IgAN (15, 27, 28). The reduction of these genera in the Nefecon group suggests a potential reversal of IgAN-associated dysbiosis. Furthermore, the abundance of certain beneficial genera, such as *Blautia (*29), was lower in the new patients and remission groups but appeared to be restored in the Nefecon group. Although the role of many specific bacteria in IgAN requires further elucidation, these collective changes are consistent with a shift towards a healthier microbial state under Nefecon treatment. Our findings on *Escherichia-Shigella*, a genus previously implicated in IgAN pathogenesis (10, 13–15, 18–20), also align with this pattern. Although the difference was not statistically significant, likely due to the small sample size, a trend of decreased *Escherichia-Shigella* abundance was noted in the Nefecon and steroid groups compared to the new patients group. This trend is consistent with a previous study reporting that the suppression of *Escherichia-Shigella* is associated with a positive response to immunosuppressive therapy (15).

The dbRDA results further supported an association between treatment and microbiota composition. In the ordination plot, the arrows denote clinical variables, with their direction indicating that samples with higher values for a given variable tend to cluster along that axis. Samples from the new patients and remission groups clustered in proximity in the ordination space, indicating a shared gut microbiota profile. In contrast, samples from the Nefecon and steroid groups exhibited greater dispersion and separated from the former clusters, suggesting that these treatments may modulate microbial community structure.

Several limitations of our study should be acknowledged. The small sample size limited the statistical power and the generalizability of our findings. Furthermore, the cross-sectional nature of our study makes it difficult to establish causal relationships between the observed microbiota changes and clinical outcomes, and our intergroup comparisons should therefore be interpreted as exploratory. Also, mechanistic understanding is limited by the absence of functional assessments, including microbial metabolites, intestinal barrier integrity, and host immune responses. Future studies with larger cohorts and longitudinal sampling are warranted to validate these preliminary observations.

## 5 Conclusion

This comparative study suggests that despite the efficacy of RAS blockers in resolving proteinuria, underlying dysbiosis may persist. Nefecon, however, may exert beneficial effects on the gut microbiota, which could contribute to its therapeutic efficacy. These findings highlight the gut microenvironment as a promising therapeutic target in IgAN and warrant further investigation into the role of gut microbiota in treatment response.

## Data Availability

The data presented in the study are deposited in the NCBI Sequence Read Archive (SRA) repository, accession number PRJNA1504449.
