# Supplementary material for: Targeted release of budesonide modulates key gut microbial abnormalities in immunoglobulin A nephropathy
Source: Front Immunol. 2026 Aug 7;17:1856526. doi: 10.3389/fimmu.2026.1856526 (PMC13493527; doi:10.3389/fimmu.2026.1856526)
Supplement: Supplementary file 1 [file Table1.docx]

Supplementary Material

**Supplementary Figure 1.** **Differentially abundant genera between the Newpts and Nefecon groups.** These nine genera are not included in the 24 genera presented in Figure 7.

**Supplementary Figure 2.** **Screening of previously reported IgAN-associated genera (Part I).** Differentially abundant genera from literature that were also detected in our study.

**Supplementary Figure 3.** **Screening of previously reported IgAN-associated genera (Part II).** Differentially abundant genera from literature that were also detected in our study.

| **ID** | **Gender** | **Age (years)** | **Scr (umol/L)** | **eGFR**  **(mL/min/1.73m^2^)** | **Proteinuria**  **(g/24h)** | **M** | **E** | **S** | **T** | **C** | **Sclerosis%** |
| --- | --- | --- | --- | --- | --- | --- | --- | --- | --- | --- | --- |
| **Newpts1** | Male | 79 | 199 | 27 | 0.1 | 0 | 0 | 1 | 0 | 0 | 27 |
| **Newpts2** | Male | 55 | 173 | 37 | 3.32 | 1 | 0 | 1 | 1 | 1 | 10 |
| **Newpts3** | Male | 45 | 93 | 85 | 1.87 | 1 | 0 | 1 | 0 | 0 | 9 |
| **Newpts4** | Male | 52 | 100 | 74 | 1.94 | 0 | 1 | 1 | 0 | 1 | 8 |
| **Newpts5** | Male | 30 | 91 | 97 | 0.75 | 0 | 0 | 0 | 0 | 0 | 0 |
| **Newpts6** | Male | 26 | 77 | 119 | 0.78 | 0 | 1 | 0 | 0 | 1 | 4 |
| **Newpts7** | Male | 41 | 88 | 94 | 0.62 | 1 | 0 | 0 | 0 | 0 | 25 |
| **Newpts8** | Female | 29 | 52 | 124 | 3.3 | 0 | 0 | 0 | 0 | 2 | 4 |
| **Remission1** | Female | 53 | 81 | 71 | 0.23 | 1 | 0 | 1 | 0 | 1 | 27 |
| **Remission2** | Male | 40 | 187 | 38 | 0.13 | 0 | 0 | 1 | 1 | 0 | 10 |
| **Remission3** | Male | 33 | 88 | 99 | 0.18 | 0 | 0 | 1 | 0 | 0 | 13 |
| **Remission4** | Male | 30 | 85 | 105 | 0.18 | 1 | 1 | 1 | 0 | 0 | 6 |
| **Remission5** | Male | 42 | 114 | 68 | 0.39 | 0 | 0 | 1 | 1 | 0 | 42 |
| **Remission6** | Male | 34 | 92 | 93 | 0.27 | 0 | 1 | 1 | 0 | 1 | 12 |
| **Steriod1** | Male | 41 | 97 | 87 | 0.68 | 1 | 1 | 1 | 0 | 1 | 7 |
| **Steriod2** | Female | 40 | 81 | 81 | 0.523 | 1 | 1 | 1 | 0 | 1 | 9 |
| **Steriod3** | Female | 38 | 170 | 34 | 0.26 | 0 | 0 | 1 | 1 | 0 | 64 |
| **Steriod4** | Female | 53 | 142 | 38 | 0.65 | 1 | 0 | 1 | 1 | 1 | 19 |
| **Steriod5** | Male | 52 | 66 | 124 | 1.22 | 0 | 0 | 1 | 0 | 0 | 21 |
| **Nefecon1** | Female | 31 | 101 | 66 | 0.6 | 0 | 0 | 1 | 0 | 0 | 32 |
| **Nefecon2** | Female | 46 | 192 | 28 | 0.85 | 1 | 0 | 1 | 1 | 0 | 69 |
| **Nefecon3** | Male | 47 | 97 | 84 | 0.68 | 0 | 0 | 1 | 0 | 1 | 47 |
| **Nefecon4** | Female | 46 | 60 | 109 | 0.48 | 0 | 0 | 1 | 0 | 1 | 23 |
| **Nefecon5** | Male | 44 | 206 | 35 | 3.46 | 1 | 0 | 1 | 1 | 1 | 12 |

**Supplementary Table1. Clinical information of the IgAN patients.** Scr, serum creatinine; eGFR, estimated glomerular filtration rate; M, mesangial hypercellularity; E, endocapillary cellularity; S, segmental sclerosis; T, interstitial fibrosis/tubular atrophy; C, crescent; Sclerois%, percentage of global glomerulosclerosis.

**Supplementary Table2. Genera and phyla detected in all samples.** C1-C9: Control group. N1-N8: Newpts group. H1-H6: Remission group. S1-S5: Steroids group. Nef1-Nef5: Nefecon group.

**Supplementary Table3. 132 differentially abundant genera across the five groups.**
